# Tractometry of the Human Connectome Project: resources and insights

**DOI:** 10.3389/fnins.2024.1389680

**Published:** 2024-06-12

**Authors:** John Kruper, McKenzie P. Hagen, François Rheault, Isaac Crane, Asa Gilmore, Manjari Narayan, Keshav Motwani, Eardi Lila, Chris Rorden, Jason D. Yeatman, Ariel Rokem

**Affiliations:** ^1^Department of Psychology, University of Washington, Seattle, WA, United States; ^2^Department of Computer Science, Universitè de Sherbrooke, Sherbrooke, QC, Canada; ^3^Department of Psychology, University of Chicago, Chicago, IL, United States; ^4^Graduate School of Education, Stanford University, Stanford, CA, United States; ^5^Department of Biostatistics, University of Washington, Seattle, WA, United States; ^6^Department of Psychology, University of South Carolina, Columbia, SC, United States

**Keywords:** brain, MRI, diffusion MRI, tractometry, Open Data, heritability, predictive modeling, data visualization

## Abstract

**Introduction:**

The Human Connectome Project (HCP) has become a keystone dataset in human neuroscience, with a plethora of important applications in advancing brain imaging methods and an understanding of the human brain. We focused on tractometry of HCP diffusion-weighted MRI (dMRI) data.

**Methods:**

We used an open-source software library (pyAFQ; https://yeatmanlab.github.io/pyAFQ) to perform probabilistic tractography and delineate the major white matter pathways in the HCP subjects that have a complete dMRI acquisition (*n* = 1,041). We used diffusion kurtosis imaging (DKI) to model white matter microstructure in each voxel of the white matter, and extracted tract profiles of DKI-derived tissue properties along the length of the tracts. We explored the empirical properties of the data: first, we assessed the heritability of DKI tissue properties using the known genetic linkage of the large number of twin pairs sampled in HCP. Second, we tested the ability of tractometry to serve as the basis for predictive models of individual characteristics (e.g., age, crystallized/fluid intelligence, reading ability, etc.), compared to local connectome features. To facilitate the exploration of the dataset we created a new web-based visualization tool and use this tool to visualize the data in the HCP tractometry dataset. Finally, we used the HCP dataset as a test-bed for a new technological innovation: the TRX file-format for representation of dMRI-based streamlines.

**Results:**

We released the processing outputs and tract profiles as a publicly available data resource through the AWS Open Data program's Open Neurodata repository. We found heritability as high as 0.9 for DKI-based metrics in some brain pathways. We also found that tractometry extracts as much useful information about individual differences as the local connectome method. We released a new web-based visualization tool for tractometry—“Tractoscope” (https://nrdg.github.io/tractoscope). We found that the TRX files require considerably less disk space-a crucial attribute for large datasets like HCP. In addition, TRX incorporates a specification for grouping streamlines, further simplifying tractometry analysis.

## 1 Introduction

The long-range connections between different brain areas that form the human macro-scale connectome are essential to the distribution and integration of information in the brain (Bassett and Sporns, 2017). Healthy brain connections are also important for mental and neurological health (Bassett and Bullmore, 2009). The Human Connectome Project (HCP) is a pioneering effort to study the structure and function of the brain macro-scale connectome. The WU-Minn-Ox consortium of the HCP pursued this effort by collecting a large dataset of 1,200 young adult twin and non-twin siblings that included extensive measurements of structural (T1-weighted and T2-weighted), functional (both with a task and without one—i.e., at “rest”) and diffusion-weighted MRI (dMRI), in addition to genotype information and behavioral testing. Some of the subjects also underwent additional electrophysiological measurements and additional MRI measurements at 7T.^1^ Rather than relying on the state of the art of MRI measurements at the time that the project was initiated, the HCP advanced the field forward, developing a large number of novel techniques for data acquisition, data processing and analysis, and created novel ways to organize and disseminate the data. This effort has generated a dataset that even now, more than a decade after the project started, stands out in its high quality and uniformity of measurement, and in the large value that the research community has drawn from it. Thus, the HCP has become a keystone dataset in human neuroscience, with more than 1,500 papers that acknowledge using the data, as of 2021 (Elam et al., 2021). Its approach serves as a source of inspiration to a large number of HCP-style follow-up studies (Glasser et al., 2016), including studies targeting life-span development (Bookheimer et al., 2019; Howell et al., 2019), and several different projects targeting specific clinical populations (e.g., Demro et al., 2021).

Measurements of dMRI in the HCP dataset leveraged several technical innovations. These included use of specialized hardware, and particularly of a strong and fast set of gradients, with a maximal gradient strength of 100 mT/m, and effective slew rate of 91 mT/m/s. Parallel imaging techniques that use multi-slice and multi-band excitation were used to accelerate the acquisition of each volume (Setsompop et al., 2012). This enabled measurements in a large number of different directions, with multiple different non-zero b-values (distributed in three shells of *b*≈1, 000*s*/*mm*^2^, *b*≈2, 000*s*/*mm*^2^, *b*≈3, 000*s*/*mm*^2^), and with a high spatial resolution of 1.25 × 1.25 × 1.25*mm*^3^. In addition to these advanced acquisition techniques, HCP developed novel processing methods to address artifacts due to motion and eddy currents, and to address geometric distortions due to susceptibility. Thus, the HCP produced data that far exceeds, in terms of spatial and angular resolution, what is possible in most clinical settings, even a decade later. Therefore, these dMRI data provide unique views of the human white matter connectome.

Tractometry analysis of dMRI data focuses on the physical properties of major white matter pathways. It uses computational tractography and anatomical constraints to delineate the locations of known anatomical tracts in dMRI data, and extracts brain white matter tissue properties along the length of each tract (Yeatman et al., 2012). Tractometry provides important information about brain tissue properties and individual differences, but for large and important datasets, such as the HCP, applying cutting-edge tractometry methods requires specialized expertise, and is also very computationally demanding. The present work enables the study of brain connections in the HCP dataset by providing tractometry results in 1,041 subjects in HCP that have completed a full set of dMRI measurements and by building a set of insights and resources based on this data. In each subject in the dataset, 24 major white matter pathways were identified using the pyAFQ software (https://yeatmanlab.github.io/pyAFQ) (Table 1). We used probabilistic tractography to delineate the tracts and diffusion kurtosis imaging (DKI) (Jensen et al., 2005) as implemented in the open-source software DIPY (https://dipy.org) (Garyfallidis et al., 2014; Henriques et al., 2021) to describe white matter tissue properties along their lengths. DKI was used because it extends diffusion tensor imaging (DTI) (Basser et al., 1994), providing a more complete assessment of diffusion by measuring the deviation of the diffusion patterns from a Gaussian distribution. In addition, in previous work, we have also shown that DKI describes the HCP dMRI data more accurately and more reliably than DTI (Henriques et al., 2021). Here, we also used an extension of DKI that models biophysical white matter tissue properties (Fieremans et al., 2011) to provide additional information about the axonal white matter fraction along the length of the major white matter pathways. The results of this processing are all provided openly through the AWS Open Data program in the Open Neurodata repository (Vogelstein et al., 2018), and we provide an example of how to access this data.

**Table 1:** Abbreviations used for the tracts saved in both the TRK and TRX format.

**Tract abbreviation**	**Formal tract name**
ATR_L	Left anterior thalamic
ATR_R	Right anterior thalamic
CST_L	Left corticospinal
CST_R	Right corticospinal
CGC_L	Left cingulum cingulate
CGC_R	Right cingulum cingulate
FP	Forceps major
FA	Forceps minor
IFO_L	Left inferior fronto-occipital
IFO_R	Right inferior fronto-occipital
ILF_L	Left inferior longitudinal
ILF_R	Right inferior longitudinal
SLF_L	Left superior longitudinal
SLF_R	Right superior longitudinal
UNC_L	Left uncinate
UNC_R	Right uncinate
ARC_L	Left arcuate
ARC_R	Right arcuate
Orbital	Orbital corpus callosum
AntFrontal	Anterior frontal callosum
SupFrontal	Superior frontal callosum
Motor	Motor corpus callosum
SupParietal	Superior parietal corpus callosum
PostParietal	Posterior parietal corpus callosum
Occipital	Occipital corpus callosum
Temporal	Temporal Corpus Callosum

We used this open dataset as a platform to examine several different aspects of the data. First, we characterized the overall distribution of tissue properties along the length of the white matter pathways that we delineated. We also used the presence of a large number of monozygotic and dizygotic twins in the sample to characterize the heritability of DKI tissue properties along the length of the tracts. Finally, we compared the predictive ability of tract profiles to other diffusion processing methods. Tract profiles of tissue properties can be used to compare different subject groups or in order to understand individual differences (Jones et al., 2005; Colby et al., 2012; Yeatman et al., 2012; Dayan et al., 2016; Richie-Halford et al., 2021). However, high-dimensional data with limited observations can challenge the accuracy of out-of-sample predictions, providing motivation to understand if there is any loss of predictive information with the dimensionality reduction provided by tract profiles. In a previous study (Rasero et al., 2021), brain-behavior correlations were assessed using the local connectome (LC) method (Yeh et al., 2016), which calculates a q-space normalized map of the density of spins between neighboring locations along tracts. The resulting feature sets from each method differ in their dimensionality—tract profiles for every standard tract results in several thousand features, while LC results in hundreds of thousands of features. In the present study, we compared the information provided by LC to the much more concise information provided in tractometry tract profiles. Open access to a standard HCP tractometry dataset will facilitate future research aimed at comparing additional methods for analysis of brain behavior correlations.

Following the long-standing tradition of the HCP, our development of HCP tractometry results provides a platform for developing and advancing new technologies. We used HCP tractometry as a platform to test TRX, a recently-proposed community-based file format that incorporates the benefits of several previously-developed file formats for tractography, and that advances several new innovative features (Rheault et al., 2022). In the present work, we used HCP tractometry to test the computational efficiency of TRX and its potential to conserve storage space, while retaining important information about tract profile features. Finally, interactive web-based visualization tools for exploring large datasets lower the barrier for fruitful interaction with these datasets, and serve as a point of entry for researchers who are considering how to use the data (Keshavan and Poline, 2019). In previous work, we developed AFQ-Browser (https://yeatmanlab.github.io/AFQ-Browser), an application that enables exploration of tractometry datasets (Yeatman et al., 2018), but the previously presented tool was limited in terms of its ability to explore the anatomical structure of each individual subject in the dataset. The recent development of the NiiVue software library enables much more facile visualization of anatomical data (Hanayik et al., 2024), including both volumetric and tractography datasets and their combination. Here, we present Tractoscope (https://nrdg.github.io/tractoscope), as the next generation of web-based tools for sharing and exploring tractometry results.

## 2 Methods

### 2.1 Data

Diffusion MRI data was collected by the Human Connectome Project (HCP), as previously described in detail (Sotiropoulos et al., 2013). Briefly, data was acquired on a 3T Siemens Skyra MRI system equipped with a 32-channel coil that was modified to accommodate gradients with *G*_*max*_ = 100*mT*/*m* (ultimately, acquisition was conducted with a *G*_*max*_ = 97.4*mT*/*m* after optimization for gradient duty cycle). Multislice echo planar imaging with mulitband excitation was acquired with a TR of 5.5 s and TE of 89 ms. Three diffusion-weighted shells were acquired: *b*≈1, 000*s*/*mm*^2^, *b*≈2, 000*s*/*mm*^2^, *b*≈3, 000*s*/*mm*^2^ and the same TR/TE was used in each. In each shell, 90 non-colinear directions were selected, to optimize coverage within and across shells (Caruyer et al., 2013), resulting in the acquisition of 190 data points in each shell, corresponding to measurements in inverse phase encoding direction (LR and LR directions) and five non-diffusion weighted acquisitions. The spatial resolution of the data was 1.25 × 1.25 × 1.25*mm*^3^.

We used data provided by HCP that had already been processed using the HCP minimal preprocessing pipelines, as previously described (Glasser et al., 2013). Briefly, intensity normalization was performed across the six acquisition series based on the non diffusion-weighted images (*b*_0_). These *b*_0_ images were also used to estimate and correct EPI distortions using the FSL “topup” tool (Andersson et al., 2003). The FSL “eddy” tool was used to correct artifacts due to eddy currents and motion (Andersson and Sotiropoulos, 2016). Gradient spatial non-linearities were computed (Bammer et al., 2003). A spatial transform was calculated between the average *b*_0_ image and the T1-weighted data using FreeSurfer's “BBRegister” algorithm (Greve and Fischl, 2009). The eddy-corrected data were transformed according to both the gradient nonlinearity correction and T1w registration into 1.25 mm structural volume space in a single step.

We analyzed data from 1,041 subjects from the HCP who had complete measurements of dMRI (i.e., where these measurements passed the HCP quality control process, and also included all 270 diffusion MRI volumes). Among these subjects, the average age was 28.7 years ± 3.7 years (standard deviation); 479/562 were male/female.

### 2.2 Tractometry analysis

We applied the pyAFQ pipeline to perform advanced tractometry analysis (Kruper et al., 2021). We used data provided by HCP that had already been pre-processed (Glasser et al., 2013; Sotiropoulos et al., 2013). Using pyAFQ, we fit constrained spherical deconvolution (CSD) and used it as the fiber orientation distribution function for probabilistic tractography implemented in DIPY (Tournier et al., 2008; Garyfallidis et al., 2014). We used symmetric normalization (SyN) (Avants et al., 2008) diffeomorphic non-linear registration to register subjects to the Montreal Neurological Institute (MNI) template (Fonov et al., 2011). We calculated the non-linear registration because the linear registration to the T1w volume that was already applied in preprocessing does not take into account more subtle local differences in brain anatomy that need to be taken into account in defining the trajectory of major white matter pathways. Twenty-four different white matter tracts were defined in template space based on a combination of inclusion and exclusion regions of interest (ROI). Sixteen are from the original AFQ templates (Wakana et al., 2007; Yeatman et al., 2012), and eight are callosal tracts (Dougherty et al., 2007). The tracts are enumerated in Table 1. The ROIs are primarily planar “inclusion” ROIs, where streamlines transecting the ROIs are assigned to be part of the bundle. However, some of the ROIs are “endpoint” ROIs, where streamlines must either start or end in the ROI, and some are “exclusion” ROIs, where streamlines cannot transect the ROI, to be assigned. The ROIs for each tract were transformed into the individual subject anatomical coordinates using the inverse of the transformation defined by SyN from the subject to the template space. Streamlines were selected from the whole-brain tractography based on whether they passed through inclusion ROIs and did not pass through exclusion ROIs for each tract. After initial selection was conducted, individual streamlines may additionally have been excluded based on whether they were extreme outliers. Streamlines were considered outliers if their Mahalanobis distance to other streamlines is greater than three standard deviations or if their length was more than five standard deviations from the mean length. This outlier exclusion was conducted over five rounds, similar to the original AFQ procedure (Yeatman et al., 2012). The diffusion kurtosis imaging (DKI) model was fit using the DIPY implementation to create the following maps of microstructural tissue properties: fractional anisotropy (FA), mean diffusivity (MD), and mean kurtosis (MK) (Henriques et al., 2021), as well as axonal water fraction (AWF) from the White Matter Tract Integrity (WMTI) model (Fieremans et al., 2011). In each tract, every streamline was resampled to 100 nodes, and tract profiles were generated by sampling the FA, MD, MK, AWF maps using these positions. The contributions of each streamline to the tract profile at each position was inversely weighted by the distance of that node from the median of the streamline positions for that node (Yeatman et al., 2012).

### 2.3 Evaluating heritability of tract profiles

The collection of data from both monozygotic (MZ) and dizygotic (DZ) twins in the HCP dataset enables an assessment of the genetic linkage, or heritability, of traits measured in the data with Haseman-Elston regression (Haseman and Elston, 1972). In this method, identity by descent in each twin pair is regressed against the square of the difference between twins in the tissue property tract profiles at every position along each tract (Equation 1):


(1) $$\begin{array}{l} {\left(Y_{ijk1}-Y_{ijk2}\right)}^{2}=\alpha +\beta \pi_{i}, \end{array}$$

where *i* is an index of the twin pair, *Y*_*ij*1_−*Y*_*ij*2_ is the difference between the two members of this twin pair in the tissue property value at position *j* (1-100) along tract *k* (1-24; Table 1). The genetic linkage π_*i*_ is assessed through the degree of identity by descent (i.e., π_*i*_ = 1.0 for MZ and π_*i*_ = 0.5 for DZ twins). Heritability of the tissue properties for position/tract *jk*, $h_{jk}^{2}$ is then estimated as (Equation 2):


(2) $$\begin{array}{l} h_{jk}^{2}=-\beta /\left(2\sigma_{jk}^{2}\right), \end{array}$$

where $\sigma_{jk}^{2}$ is the variance of the squared difference ${\left(Y_{ijk1}-Y_{ijk2}\right)}^{2}$ across *i*.

### 2.4 Evaluating brain-behavior correlations in tractometry data

We used tractometry-generated tract profiles for every tract as input features to a regularized predictive model to investigate the brain-behavior correlations of tractometry and a variety of cognitive and non-cognitive phenotypes. Each phenotype was predicted individually using a LASSO regularized linear model where the input features were the 100 node-level FA, MD, MK and AWF measurements from each of 24 tracts. LASSO regularized linear models remove unimportant features by shrinking the model weights of coefficients to zero (Tibshirani, 1996). In addition to the LASSO regularized models, the inherent grouping of tract profiles into tracts and tissue properties provides an opportunity to use models that exploit such groupings, such as Sparse Group LASSO (SGL) (Simon et al., 2013; Richie-Halford et al., 2021). In addition to the shrinking of individual features, SGL shrinks entire groups toward zero, eliminating both uninformative features and groups. As a comparison, we also created LASSO models using a different tissue property description, the local connectome (Yeh et al., 2016). This approach calculates a q-space normalized map of the density of spins between neighboring locations along tracts, producing a much larger number of features (128,894 features for each subject in LC, compared to 9,600 tract profile features). These features were also used as input features to a LASSO regularized model. A nested 5-fold cross-validation procedure was used to determine the level of regularization that was used, for fitting and for evaluation. To evaluate the reliability of our models, each model was ran 100 times, using different splits for cross validation (CV). Because the dataset contains familial relationships, cross-validation was done with respect to family, such that individuals within the same family were always assigned to the same fold. Models were evaluated on their predictive ability using the out-of-sample coefficient of determination *R*^2^ and on reliability using 95% confidence intervals of their model weights across the different CV splits.

### 2.5 TRX and TRK comparison

By default, pyAFQ generates outputs using the popular TrackVis file format (TRK) (Wang et al., 2007). However, this format does have limitations for our application. First of all, the format can not represent multiple tracts in a single file, requiring many files to represent all tracts. Second, TRK files are large and slow to read, both of which impact online data visualization and analyses. Therefore, to test the new TRX format and compare it to TRK performance, the full and segmented tractograms generated during processing by pyAFQ were converted from TRK format to TRX format (Rheault et al., 2022). The data for both formats have been made available on the Open NeuroData AWS bucket. The TRX format allows users to set the data type of tractogram coordinates/vertices, and we chose to save the tractograms as half floats. We also used TRX's built-in zip compression option. We re-calculated tract profiles from the TRK and TRX files while profiling for time and memory usage, in order to compare their performance.

### 2.6 Tractoscope

We developed a web-based application to visualize individual subject data from the HCP. The application was built using the Vue JavaScript framework and the NiiVue package (Hanayik et al., 2024). The application connects directly to the AWS bucket and uses the REST API provided by AWS buckets to query for the presence of expected files and to render the files into the browser window. The application leverages the Pinia datastore library (https://pinia.vuejs.org/) to encapsulate and manage the large amounts of data that the application needs to operate. The source code is managed on an open-source GitHub repository (https://github.com/nrdg/tractoscope) and the application is deployed using npm running on the netlify continuous delivery platform to the GitHub Pages web service.

## 3 Results

### 3.1 Openly available pyAFQ HCP derivatives

**Figure 1:**
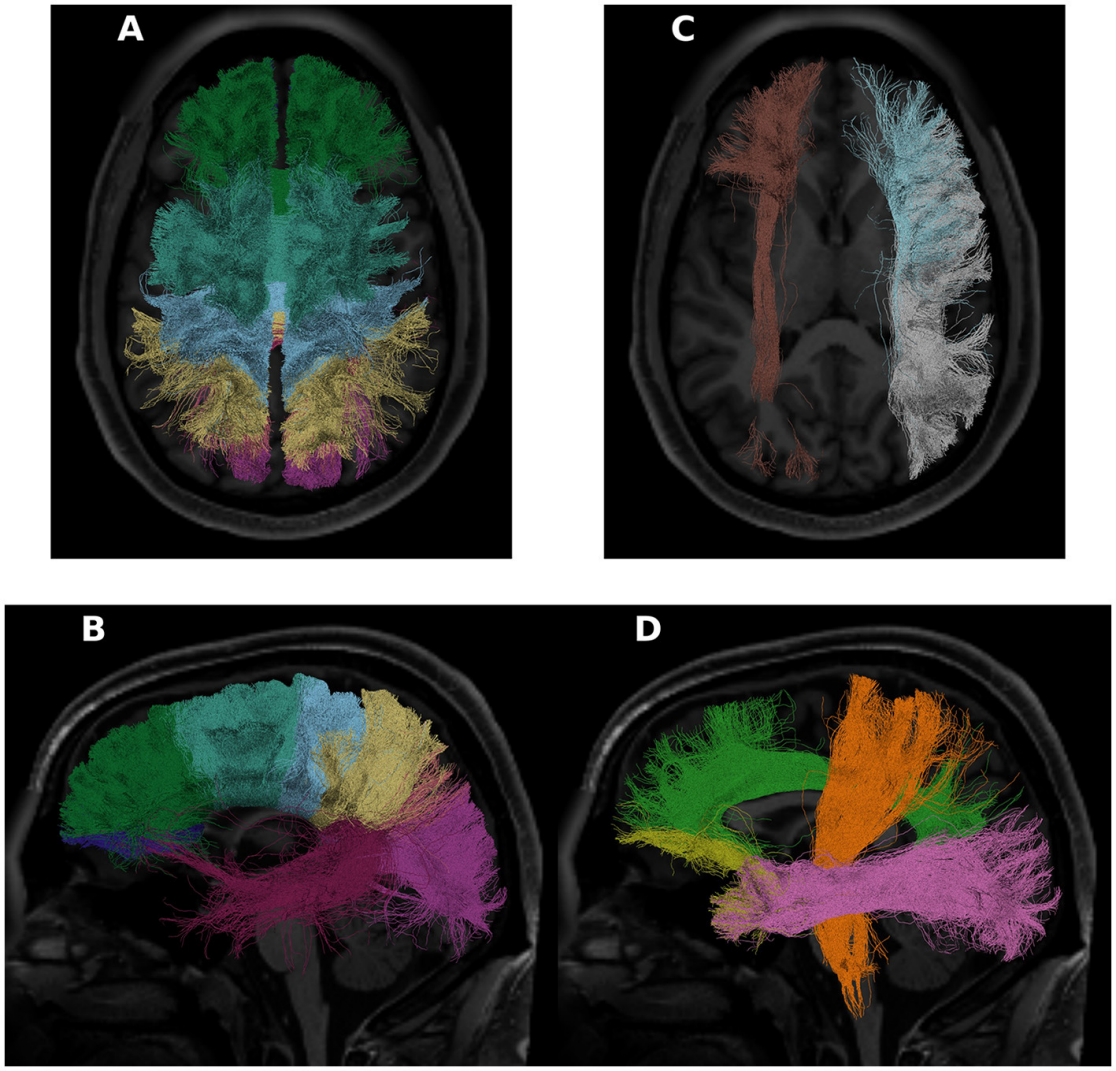
Some of the tracts recognized in a randomly chosen HCP subject (subject ID: 550439). On the left, in **(A, B)**, we see the 8 callosal tracts visualized. In **(C)**, we see the left inferior frontal occipital fasciculus in brown, and the right arcuate and superior longitudinal fasciculus in blue and white, respectively. In **(D)**, the cortiscopinal tract is shown in orange, the cingulum is shown in green, the uncinate is shown in yellow, and the inferior longitudinal fasciculus is shown in pink. For this panel, all shown tracts are from the left hemisphere. In all panels, the subject T1 is used as the background.

**Figure 2:**
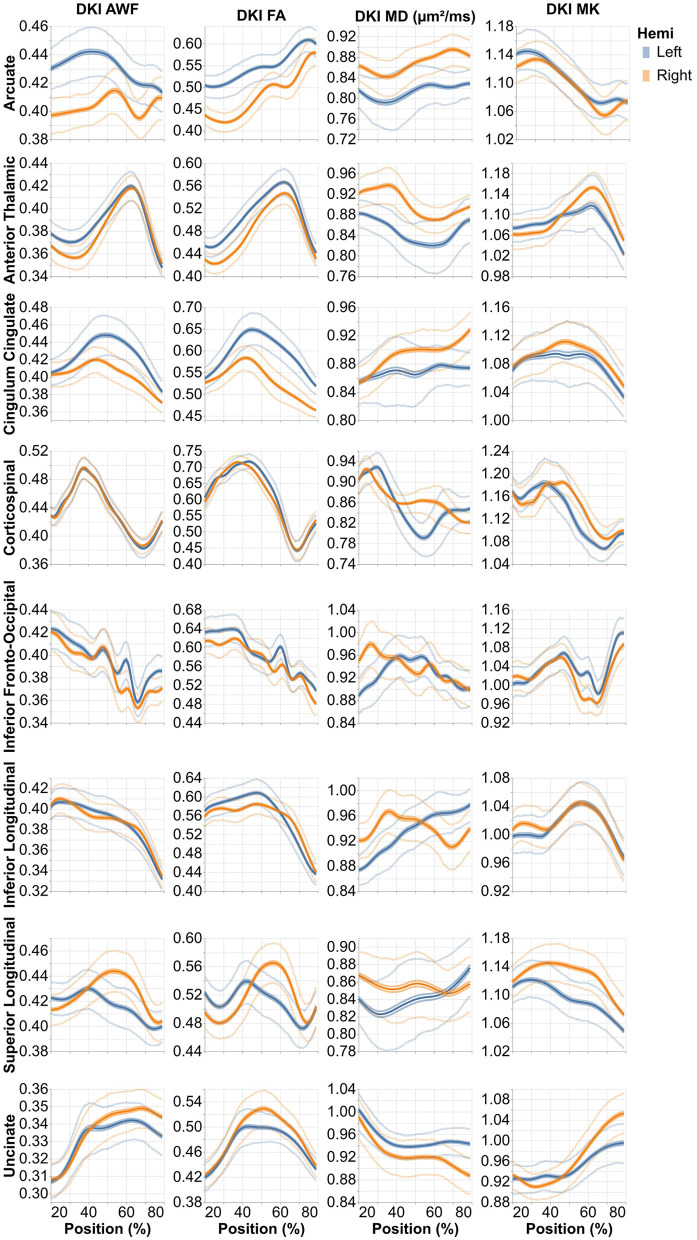
Average profiles from the 16 standard non-callosal pyAFQ tracts for all HCP subjects. The *x*-axis encodes position along the given bundle, discretized into 100 positions per bundle. The thin lines that tightly hug the average profile indicate the 95% confidence interval, and they are often hard to see as they closely follow the mean, due to the large sample size. The thinner lines indicate the interquartile range. Different rows correspond to different tracts, with color showing the hemisphere. The different columns show different tissue properties, from left to right: axonal water fraction, fractional anisotropy, mean diffusivity, and mean kurtosis.

**Figure 3:**
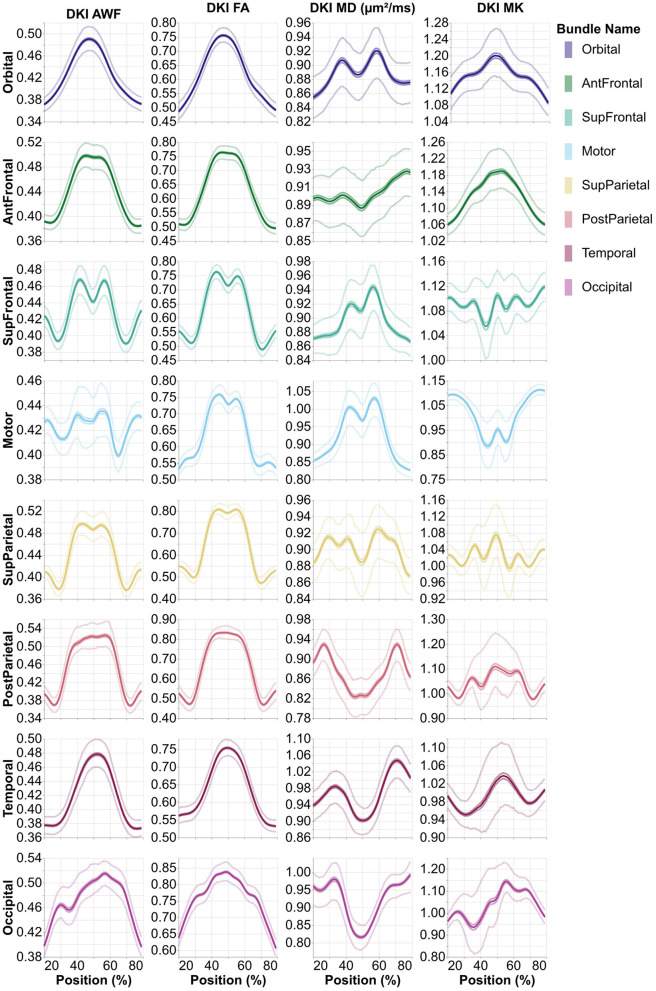
Average tract profiles from the eight absence of any commercial or financial relationships callosal pyAFQ tracts for all HCP subjects. The *x*-axis encodes position along the given bundle, discretized into 100 positions per bundle. The thin lines that tightly hug the average profile indicate the 95% confidence interval, and they are often hard to see as they closely follow the mean, due to the large sample size. The thinner lines indicate the interquartile range. Different rows and colors correspond to different subdivisions of the callosal tracts. The different columns show different tissue properties, from left to right: axonal water fraction, fractional anisotropy, mean diffusivity, and mean kurtosis.

All of the derivatives generated by pyAFQ to perform each of the steps in processing have been made available through the AWS Open Data programs' Open Neurodata bucket (Vogelstein et al., 2018). The results of tract recognition on a single randomly selected subject (subject ID: 550439) is shown in Figure 1. The average tract profiles from all subjects for all tracts and tissue properties are shown in Figures 2, 3.

The results can be accessed using the Amazon Web Services Command Line Interface (AWS CLI; https://aws.amazon.com/cli/) at the following S3 address: s3://open-neurodata/rokem/hcp1200/. The dataset is organized using principles adapted from the Brain Imaging Data Structure (BIDS), a standard for organizing and describing neuroimaging data (Gorgolewski et al., 2016), to facilitate easy access and exploration of the data, and interoperability with other datasets. Detailed examples of data access using the AWS CLI and the boto3 Python library are provided in the Supplementary material.

### 3.2 Heritability of tract profiles of tissue properties

**Figure 4:**
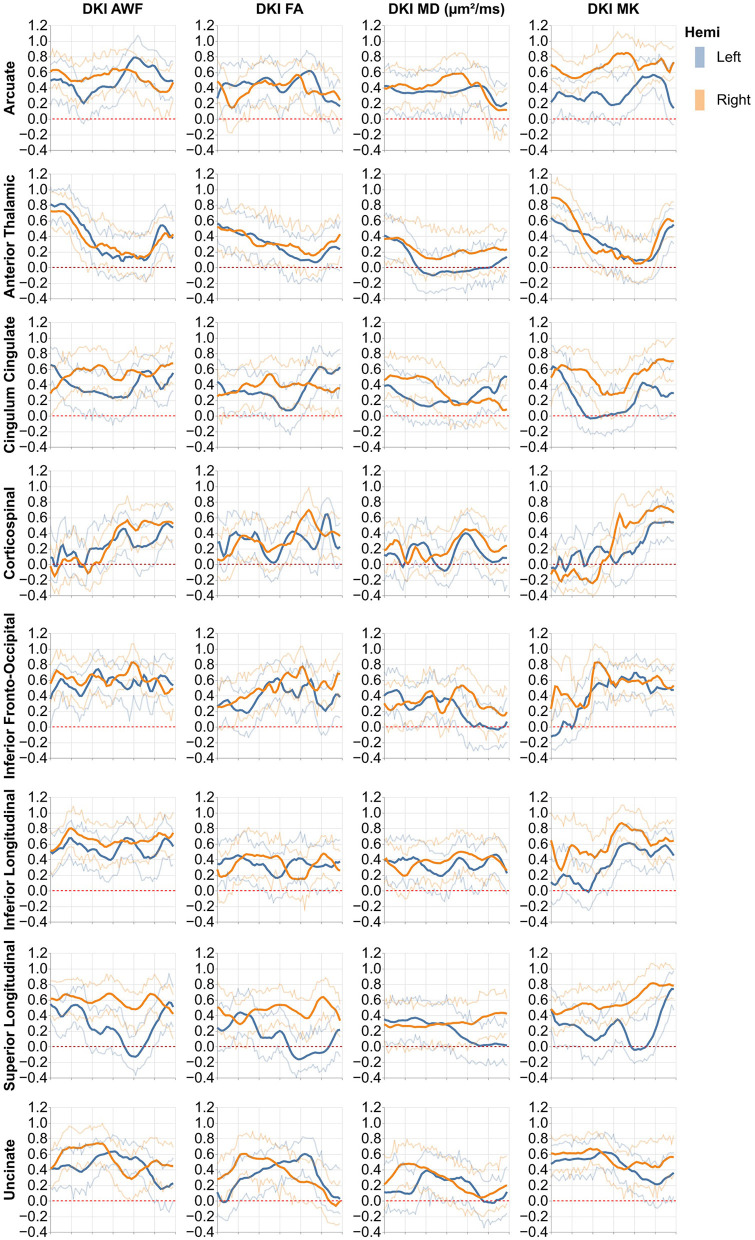
Heritability profiles from the 16 standard non-callosal pyAFQ tracts for all HCP subjects. The *x*-axis encodes position along the bundle. Thin lines indicate 95% confidence interval. Different rows correspond to different tracts, with color showing the hemisphere. The different columns show different tissue properties, from left to right: axonal water fraction, fractional anisotropy, mean diffusivity, mean kurtosis.

**Figure 5:**
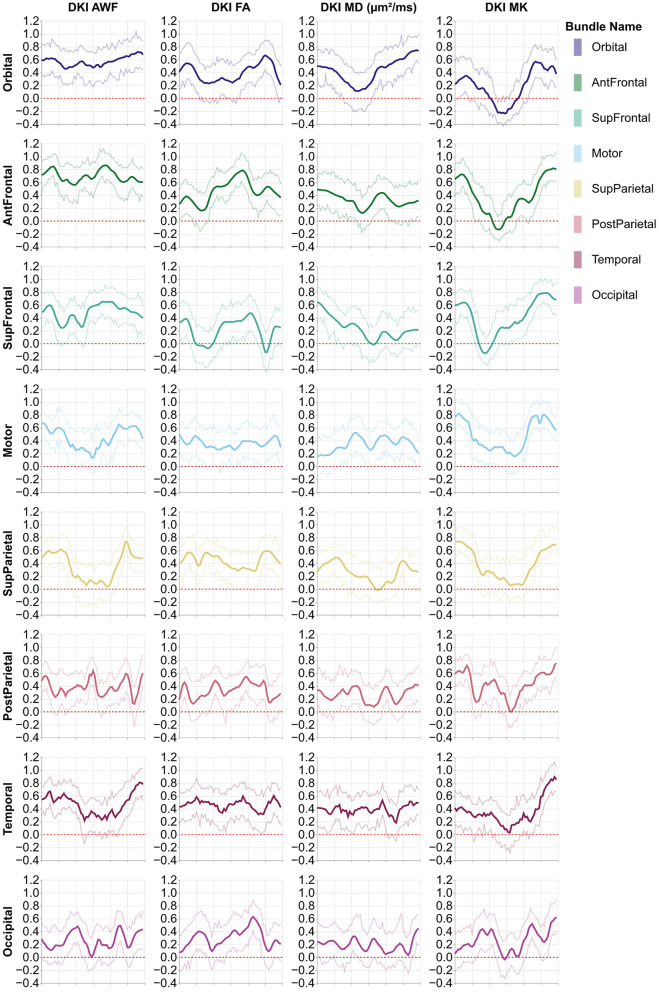
Heritability profiles from the eight callosal pyAFQ tracts for all HCP subjects. The *x*-axis encodes position along the bundle. Thin lines show the 95% confidence interval. Different rows and colors correspond to different subdivisions of the callosal tracts. The different columns show different tissue properties, from left to right: axonal water fraction, fractional anisotropy, mean diffusivity, mean kurtosis.

The heritability of tract profiles varies between tissue properties, tracts, and within each tract (Figures 4, 5). Averaging across all tracts and positions along the tracts, the heritability of the different tissue properties is: FA: *h*^2^ = 0.33 ± 0.17, MD: *h*^2^ = 0.29 ± 0.15, MK: *h*^2^ = 0.42 ± 0.25, AWF: *h*^2^ = 0.47 ± 0.2 (standard deviations across tracts and positions are reported). In most cases, we observe some symmetry across the midline, mirroring the laterality of tissue properties observed in Figures 2, 3, although this symmetry is less clear than with the tissue properties themselves. A notable exception to this symmetry is in the heritability of MK in the arcuate fasciculus, which is substantially lower in the left hemisphere than in the right hemisphere.

### 3.3 Accuracy and reliability of brain-phenotype models based on tract profile features

**Figure 6:**
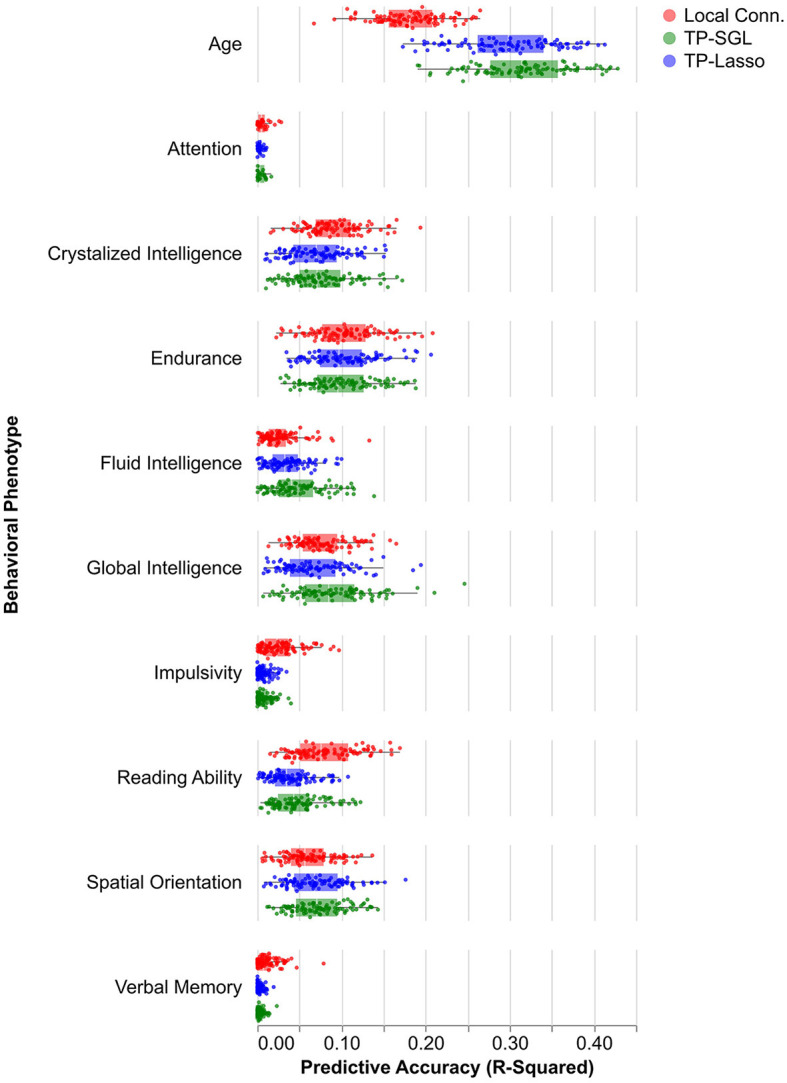
Predictive model performance by phenotype. Box and whisker plots show the distribution model accuracies by model type and input feature. Boxes show the middle 50% of accuracy values (quantified by using out of sample *R*^2^), and each point is one model run.

Regularized regression models were used to assess brain-phenotype correlations (Figure 6). Variance explained (*R*^2^) was assessed as a measure of the accuracy of the correlations, using cross-validation to mitigate the potential for overfitting within the data used for fitting. Variablility of this estimate was assessed using bootstrapping. For both tractometry and LC features, accuracy across a range of phenotypes varies between almost no predictive accuracy for all models (e.g., Attention - LC: *R*^2^ = 0.0064 95% CI [0.00010, 0.030], SGL: 0.0044 [0.00011, 0.013], LASSO: *R*^2^ = 0.0033 [0.00012, 0.010]) and moderate predictive accuracy (e.g., Age - LC: *R*^2^ = 0.18 [0.10, 0.26], SGL: *R*^2^ = 0.31 [0.21, 0.42], LASSO: *R*^2^ = 0.30 [0.19, 0.39]). Though there are nominal differences between LC and tract profile predictions in some phenotypes (e.g., Age and Reading Ability), we found no significant differences in accuracy or reliability of models that used the two methods to derive features for predictive modeling.

**Figure 7:**
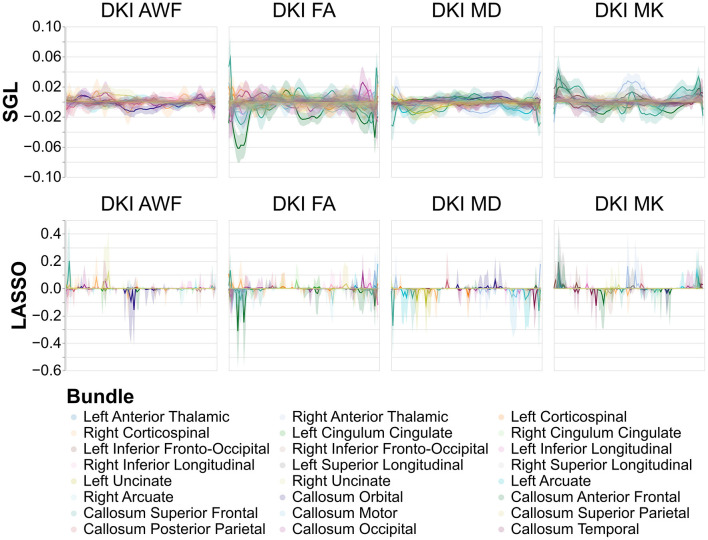
Model weights across nodes for tract profile models predicting age. The *x*-axis encodes position along the bundles, discretized into 100 positions per bundle. Solid lines show the mean model weight across bootstraps for every tract, across every node, and the shaded area show the 95% confidence intervals of the model weights. Comparing LASSO and SGL models, the model weights assigned to each node are more consistent for SGL models and model weights are spread between adjacent nodes in a tract rather than to individual nodes in each tract. The y-axis differs between SGL and LASSO panels to show the patterns of node-by-node model weights in SGL better.

While model accuracy did not vary significantly by model choice (Table 2, the reliability of the model weights for LASSO and SGL models did) (Table 3). Across phenotypes, LASSO tended to assign high model weights to individual nodes, with large variances across bootstraps. In contrast, SGL assigned smaller model weights to adjacent nodes within tracts, with much smaller variances in model weights across bootstraps (Figure 7). This pattern occurs across all phenotypes (Supplementary Figures S1–S4).

**Table 2:** Variances of SGL and LASSO model weights for each phenotype across tracts.

	**SGL**	**LASSO**
Age	5.7e-03	1.5e-02
Crystalized intelligence	8.5e-03	3.0e-02
Fluid intelligence	7.4e-03	2.4e-02
Global intelligence	1.2e-02	3.6e-02
Impulsivity	5.4e-05	1.8e-04
Endurance	7.4e-03	2.8e-02
Verbal memory	8.1e-04	3.4e-03
Reading ability	9.1e-03	2.6e-02
Attention	3.5e-06	2.6e-05
Spatial orientation	3.4e-03	1.1e-02

**Table 3:** Average accuracy for each phenotype and model.

	**LASSO**	**LC**	**SGL**
Age	3e-01	1.8e-01	3.1e-01
Crystalized intelligence	7.2e-02	9e-02	7.6e-02
Fluid intelligence	3.5e-02	2.7e-02	4.8e-02
Global intelligence	6.8e-02	7.7e-02	8.9e-02
Impulsivity	7.1e-03	2.7e-02	8.2e-03
Endurance	1e-01	1e-01	1e-01
Verbal memory	3.3e-03	1.2e-02	3.7e-03
Reading ability	3.9e-02	8.1e-02	4.8e-02
Attention	3.3e-03	6.4e-03	4.4e-03
Spatial orientation	7e-02	6.1e-02	7.2e-02

### 3.4 TRX provides a storage-efficient file format for tractometry data

**Figure 8:**
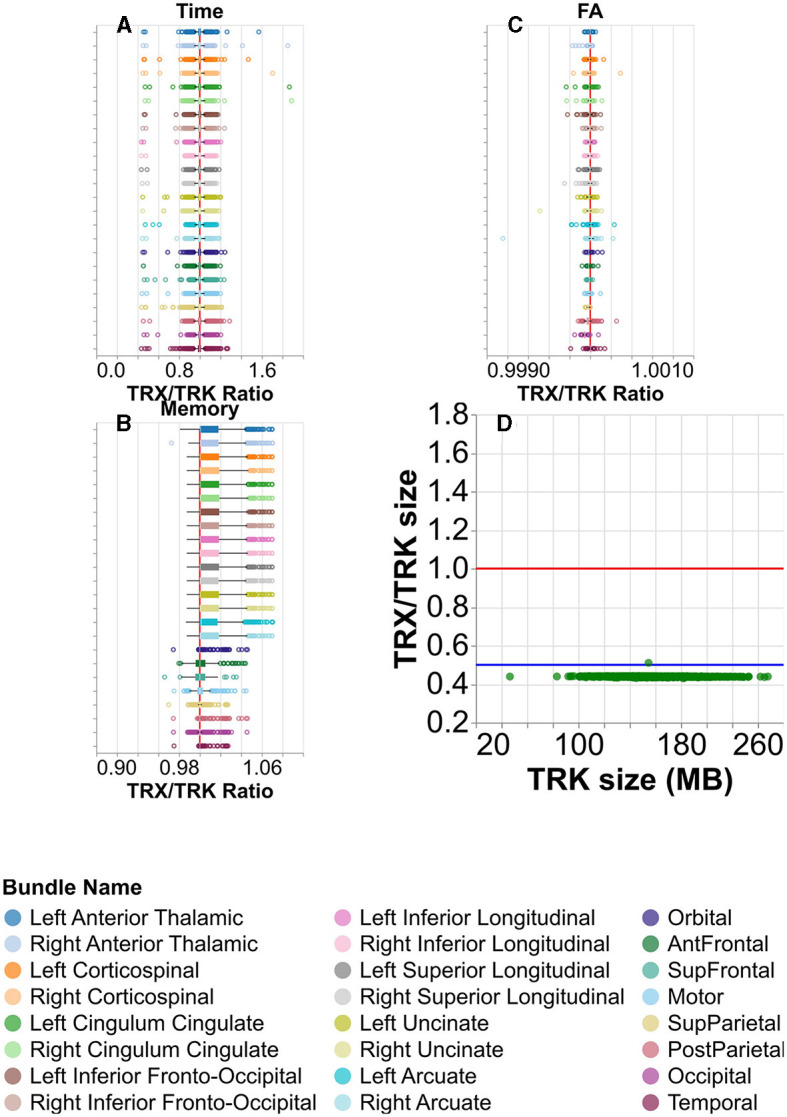
Comparing the TRK and TRX file formats. **(A)** Box and whisker plots for visualizing the distribution of the ratio of times taken to calculate tract profiles, per subject. Here, higher values would indicate it took longer to calculate tract profiles using TRX than TRK. There is a vertical red line at ratio = 1. The color/row corresponds to the tract. **(B)** Similar plot showing the memory taken to calculate tract profiles, and **(C)** the mean FA calculated. Note that in **(A–C)**, the median tightly hugs the ratio = 1 line. **(D)** The ratio of the TRX and TRK disk space size is shown for each subject in green. There is again a red line at ratio = 1, but here there is also a blue line at ratio = 0.5. Notice that the TRX/TRK size per subject in green is always near or below the blue ratio = 0.5 line.

To assess the performance of the TRX file format, we calculated tract profiles from each of the tracts using the data that was stored in the TRX file format, and calculated the ratio of the elapsed time for TRX/TRK. Performance did not susbstantially differ between the file formats (Figure 8A), except in some cases where calculation of profiles from TRX was substantially faster than with TRK. Similarly, memory usage of TRK and TRX are very similar (Figure 8B). A similar ratio was computed for the FA along the length of the tracts (Figure 8C). Despite the decreased numerical precision, and the large substantial decrease in the file sizes on disk, which often exceed a factor of 0.5X (Figure 8D), all differences in the tract profiles were smaller than 0.1%.

### 3.5 A browser-based application for exploring the HCP tractometry results

**Figure 9:**
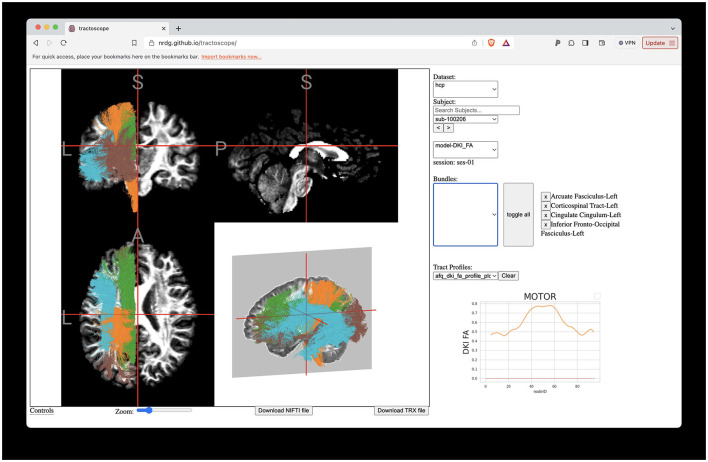
Interactive visualization of tractometry results with Tractoscope. Tractoscope is a web application designed to enable interactive exploration of results of pyAFQ processing. The application uses the NiiVue library to load data from the TRX file format. The implementation of streamline groups within TRX allows selection of different tracts. Here, we show the arcuate fasciculus, corticospinal tract, cingulum cingulate all in the left hemisphere of subject 550,436, also shown in Figure 1.

Evaluating tractometry results and viewing them without downloading any data is possible using the Tractoscope web app. Tractoscope was implemented to work with both TRK and TRX file formats, allowing users to easily explore and visualize tractography files in the HCP dataset, as well as other datasets that comply with a similar BIDS-inspired data layout. The tool is available publicly (https://github.com/nrdg/tractoscope). Any pyAFQ-compliant dataset hosted on AWS S3 buckets can be connected to the existing application with minimal configuration changes, by adding an entry to a datasets.json file. Once the AWS S3 bucket is configured to be publicly available and has HTTPS enabled, Tractoscope will be able to connect to it and visualize the dataset. The application currently enables visualizations of both the HCP dataset described here (Figure 9), as well as another dataset: the HBN-POD2 dataset, previously described in Richie-Halford et al. (2022).

## 4 Discussion

The open availability of datasets like HCP promotes collaborative studies and enhances methodological approaches. This tractometry analysis of HCP diffusion MRI data using pyAFQ and its visualization through Tractoscope exemplifies the practical benefits of accessible data. This approach facilitates a broad range of research possibilities, where different groups can use the tissue properties we share to get a more detailed understanding of white matter pathways, which are crucial for studies on neurological disorders, brain development, and cognitive functions. Some of the potential uses of the resources that we have created include: (i) as a normative sample, to be compared to various patient populations, (ii) integration with the other data that was collected by HCP in the same subjects (e.g., functional MRI measurements), (iii) further exploration of the relationships between white matter tissue properties and other phenotypic measurements, and (iv) as an educational resource for learning about the structure of human brain white matter.

The granular approach of tractometry potentially enables a more nuanced understanding of white matter variation. Additionally, by focusing on known tracts, the results of tractometry have been shown to be reliable across scans and robust to choice of model (Kruper et al., 2021). To improve interoperability between this dataset and others, we used the BIDS standard as inspiration for organizing and describing the data (Gorgolewski et al., 2016). BIDS is structured to improve the accessibility, organization, and ease of sharing complex brain imaging datasets. It employs a consistent naming scheme and directory structure, making it easier for researchers to store, analyze, and share their data with others in the field.

Analysis methods focus on various aspects of dMRI data. For example, many analysis approaches focus on generating connectivity matrices, or graphs. Connectivity results from the HCP dataset have already been published (Kiar et al., 2018). We provide a complement here, using tractometry, which allows for the evaluation of diffusion characteristics along the lengths of known tracts. Similar, tractometry-based analysis results for a subset of HCP subjects have been published as a part of larger data releases containing subjects from multiple datasets (Avesani et al., 2019; Lerma-Usabiaga et al., 2020; Hayashi et al., 2023). Here, we provide tractometry results for all subjects in HCP that have a complete dMRI acquisition. We also provide an initial characterization of population-level tract profiles in Figure 2. This characterization replicates previously known properties of human brain tract profiles. For example, there is a substantial lateralization of tissue properties in the arcuate fasciculus compared to other tracts, which is known feature of this tract (Bain et al., 2019).

### 4.1 The heritability of tract profiles

Brain structure and function has a substantial genetic component. Heritability assesses the amount of variance within a studied trait that can be explained by genetic differences. Because of their known shared genetic background, twin pairs are often studied to assess heritability. The HCP was designed with this in mind, recruiting 149 MZ and 94 DZ twin pairs (138 MZ pairs and 75 DZ pairs were included in our heritability analysis, because of missingness of DWI data in some participants). Previous research has already demonstrated that DTI-derived tissue properties are heritable at the level of tract averages both in the HCP (Kochunov et al., 2015; Gao et al., 2021), as well as in other datasets (Gustavson et al., 2019). In a few cases, heritability of DTI metrics was also assessed along the length of tracts (Lee et al., 2015). In line with these previous findings, we also found that DKI metrics can have substantial heritability up to approximately *h*^2^ = 0.9 for the DKI-specific metrics (MK and AWF) and slightly lower for metrics that are estimated in both DTI and DKI (FA and MD, which both do not exceed *h*2 = 0.8). Higher heritability seems to correspond to smaller error bars in the tract profiles, suggesting that heritability of a white matter tissue property is easier to discern when the signal is more reliably measured. The spatial variability of heritability across the length of the tracts is notable and mirrors to some extent the spatial variability of tract profiles of tissue properties. Variability in the heritability of tissue properties themselves may reflect interactions with other parts of the tissue, or different sensitivity of portions of the tracts to environmental or genetic factors.

### 4.2 Comparing tract profiles and local connectome

One of the promises of the large-scale data collection of the HCP was that the data would illuminate individual variability in a variety of behavioral measures and differences in cognitive abilities. There are a variety of different ways to assess brain-behavior correlations that are at the foundation of establishing the brain basis of individual differences. Here, we assessed the information that is available in white matter tract profiles using regularized regression approaches. As a baseline for comparison, we used features of the white matter extracted using the local connectome (LC) approach (Yeh et al., 2016). We found that both tract profiles and local connectome had small predictive skill for most phenotypes, with nominal but insignificant differences in predictive accuracy of models using tract profiles or LC as their input features (Figure 6). In line with previous literature, we found that phenotypes varied by their ability to be predicted regardless of input features, with some phenotypes like attention, verbal memory, and impulsivity having predictive accuracies near zero (Rasero et al., 2021; Roy et al., 2024). Other phenotypes, like age, had average *R*^2^ values around 0.30 for all models. Though SGL and LASSO did not differ in terms of their average accuracy, they differ substantially in terms of the variability in their feature selection properties. SGL provides much smoother and less variable selection of features.

Taken together this set of results suggests that tractometry of the human white matter extracts much of the useful information about individual differences that is present in the LC method, but the number of features is smaller by approximately an order of magnitude. This indicates that tractometry dramatically reduces the dimensionality of dMRI data, while preserving many of the features that are relevant to individual differences, to the extent that those are reflected in brain white matter tissue properties.

### 4.3 Comparing TRK and TRX

The availability of comprehensive and accessible data resources is instrumental in driving forward research in understanding brain function in health and disease. File formats and standards for storing scientific data are an important key component of the cyberinfrastructure used to disseminate and reuse scientific results, as intended here. The TRX format is a recent proposal to improve storage and access to datasets of computational tractography results (Rheault et al., 2022). The use of the TRX file format should help address the challenges of efficiently managing large neuroimaging datasets that contain such results.

Our study includes a performance comparison between TRK and TRX formats in profiling the tracts that we delineated in HCP. From Figures 8A, B, we see that the means are centered on the vertical red line, indicating that the time and memory required for calculation of tract profiles using TRX are comparable to those using TRK. From Figure 8C, we see that the differences in the resulting profiles are typically much smaller than 0.01%, with one outlier having a difference of approximately 0.01%. Additionally, TRX's integrated zip functionality and flexible data saving options enable more efficient use of disk space for storing tractograms, providing a potential for more than 2X improvement in storage, with almost no loss in information. Furthermore, the use of TRX's built-in grouping feature for segmented tractograms offers a more convenient approach compared to TRK to manage results of tractometry analysis. In TRK, segmented tracts typically necessitate additional files for storing tract identification metadata, whereas TRX simplifies this process, enhancing the efficiency of data management in neuroimaging studies.

### 4.4 Visualizing the data with Tractoscope

We developed Tractoscope, a NiiVue-based web-viewer for neuroimaging data that allows users to visualize large datasets hosted on the cloud. Tractoscope enables visualization and exploration of cloud-hosted pyAFQ-processed datasets. Tractoscope is built to work with the Amazon Web Services API, which allows it to interact dynamically with datasets that comply with the structure expected for outputs of the pyAFQ software. This significantly decreases the amount of work developers would have to do to connect the tool to future datasets. The tool is also highly configurable, allowing developers to select which scans and tracts should be made available to the user for selection through the application graphical user interface. The tool also has the ability to display tract profiles, such as those generated by pyAFQ, so long as those are stored in the graphical output format that pyAFQ generates per default. The result is a user-friendly, configurable website that can display any and all structural and diffusion imaging for datasets in the pyAFQ output format. If available, Tractoscope uses TRX files due to their increased efficiency, but it is still compatible with datasets that use TRK files.

Tractoscope demonstrates that the development of standard ways to represent large datasets facilitates the development of a wide range of standards-compliant applications, which can be universally applied to any dataset formatted according to the standard (Pestilli et al., 2021). By doing so, we ensure compatibility and interoperability across various research tools and datasets, significantly enhancing the efficiency and scope of neuroimaging research. pyAFQ operates according to these principles, as does Tractoscope. For example, Tractoscope already also visualizes subjects from the Healthy Brain Network (Alexander et al., 2017; Richie-Halford et al., 2022), in addition to HCP tractometry.

## Data availability statement

The datasets presented in this study can be found in online repositories. The names of the repository/repositories and accession number(s) can be found below: https://registry.opendata.aws/open-neurodata/.

## Ethics statement

Ethical approval was not required for the current study in accordance with the local legislation and institutional requirements, as the study used publicly available deidentified human neuroimaging datasets, and written informed consent was obtained by the Human Connectome Project for data collection. Additional written informed consent to participate in this study was not required from the participants or the participants' legal guardians/next of kin in accordance with the national legislation and the institutional requirements.

## Author contributions

JK: Conceptualization, Data curation, Formal analysis, Funding acquisition, Investigation, Methodology, Software, Visualization, Writing—original draft, Writing—review & editing. MH: Conceptualization, Formal analysis, Funding acquisition, Investigation, Visualization, Writing—original draft, Writing—review & editing. FR: Methodology, Software, Writing—review & editing. IC: Software, Writing—original draft, Writing—review & editing. AG: Software, Writing—original draft, Writing—review & editing. MN: Formal analysis, Methodology, Software, Writing—review & editing. KM: Methodology, Writing—review & editing. EL: Methodology, Funding Acquisition, Writing—review & editing. CR: Software, Funding Acquisition, Writing—review & editing. JY: Conceptualization, Funding acquisition, Writing—review & editing. AR: Conceptualization, Data curation, Formal Analysis, Funding acquisition, Investigation, Project administration, Writing—original draft, Writing—review & editing.

## Footnotes

^1^This consortium was based on a collaboration between groups at Washington University, the University of Minnesota, and Oxford University; for brevity, we will refer to this consortium as “HCP” henceforth, acknowledging that another important consortium, the MGH-UCLA consortium, pursued a different and also important approach (McNab et al., 2013).
