# Supplementary material for: Tractometry of the Human Connectome Project: resources and insights
Source: Front Neurosci. 2024 Jun 12;18:1389680. doi: 10.3389/fnins.2024.1389680 (PMC11199395; doi:10.3389/fnins.2024.1389680)
Supplement: Supplementary file 1 [file Data_Sheet_1.PDF]

Supplementary Material

1 SUPPLEMENTARY CODE EXAMPLES

1.1 Examples of HCP tractometry data access

The derivatives generated for a single subject are located at:

```
s3://open-neurodata/rokem/hcp1200/afq/sub-550439/ses-01/
```

and can be listed using the AWS CLI:

```
aws s3 ls s3://open-neurodata/rokem/hcp1200/afq/sub-550439/ses-01/
```

or copied locally with:

```
aws s3 cp s3://open-neurodata/rokem/hcp1200/afq/sub-550439/ses-01/sub
    -550439_dwi_space-RASMM_model-CSD_desc-prob_tractography.trk ./sub
    -550439_tractography.trk
```

The tract profiles for this subject can be downloaded with:

```
aws s3 cp s3://open-neurodata/rokem/hcp1200/afq/sub-550439/ ses-01/
    sub-550439_dwi_space-RASMM_model-CSD_desc-prob-afq_profiles.csv ./
    sub-550439_profiles.csv
```

Python programs can be written to automate this procedure and integrate it with other processing:

```
import boto3
client = boto3.client('s3')
subject_id = "550439"
remote_trk_fname = (
    "rokem/hcp1200/afq/sub-550439/ses-01/"
    f"sub-{subject_id}_dwi_space-RASMM_model-CSD"
    "_desc-prob_tractography.trk")
client.download_file(
    "open-neurodata",
    remote_trk_fname,
    "./sub-550439_tractography.trk")
```

1.2 Examples Tractoscope data configuration

The following is an example of the information that should be added to the `datasets.json` file

```
"hcp": {
    "bucket": "open-neurodata",
    "prefix": "rokem/hcp1200/afq/",
    "participantsSize": 2000,
```

```
    "scans": [
      "model-DKI_FA",
      "B0",
      ...]
    ],
    "bundles": [
      "AnteriorFrontal",
      "ArcuateFasciculus-Left",
      ...]
    "trxFile": {
      "fileName": "clean_tractography"
    }
  },
```

2 SUPPLEMENTARY FIGURES

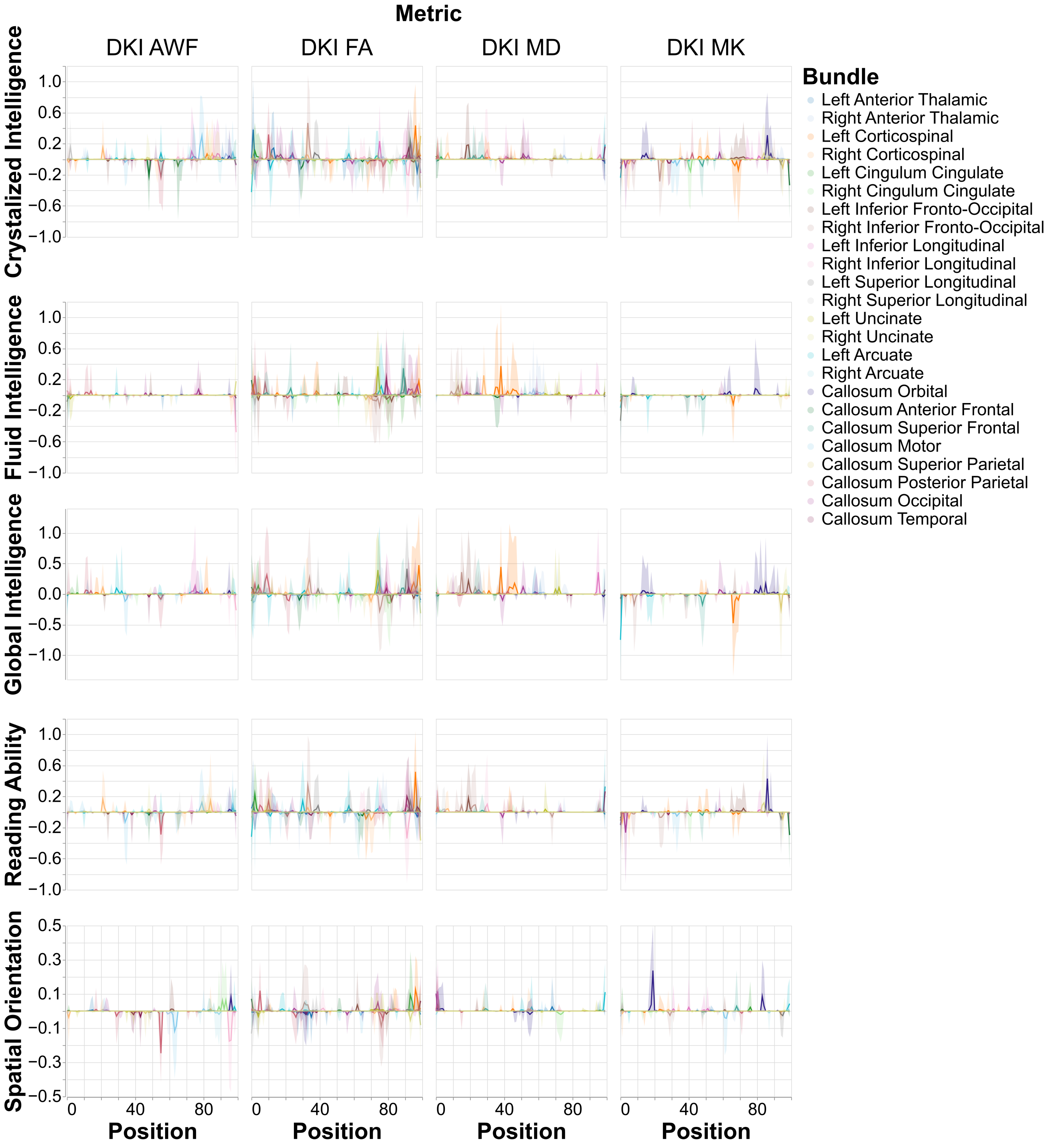

Figure S1. LASSO weights for prediction of the first five phenotypes, trained using tract profiles. Solid lines show the mean model weight across bootstraps for every tract, across every node, and the shaded area show the 95% confidence intervals of the model weights. Different columns show different white matter tissue properties, while the columns show five different phenotypes.

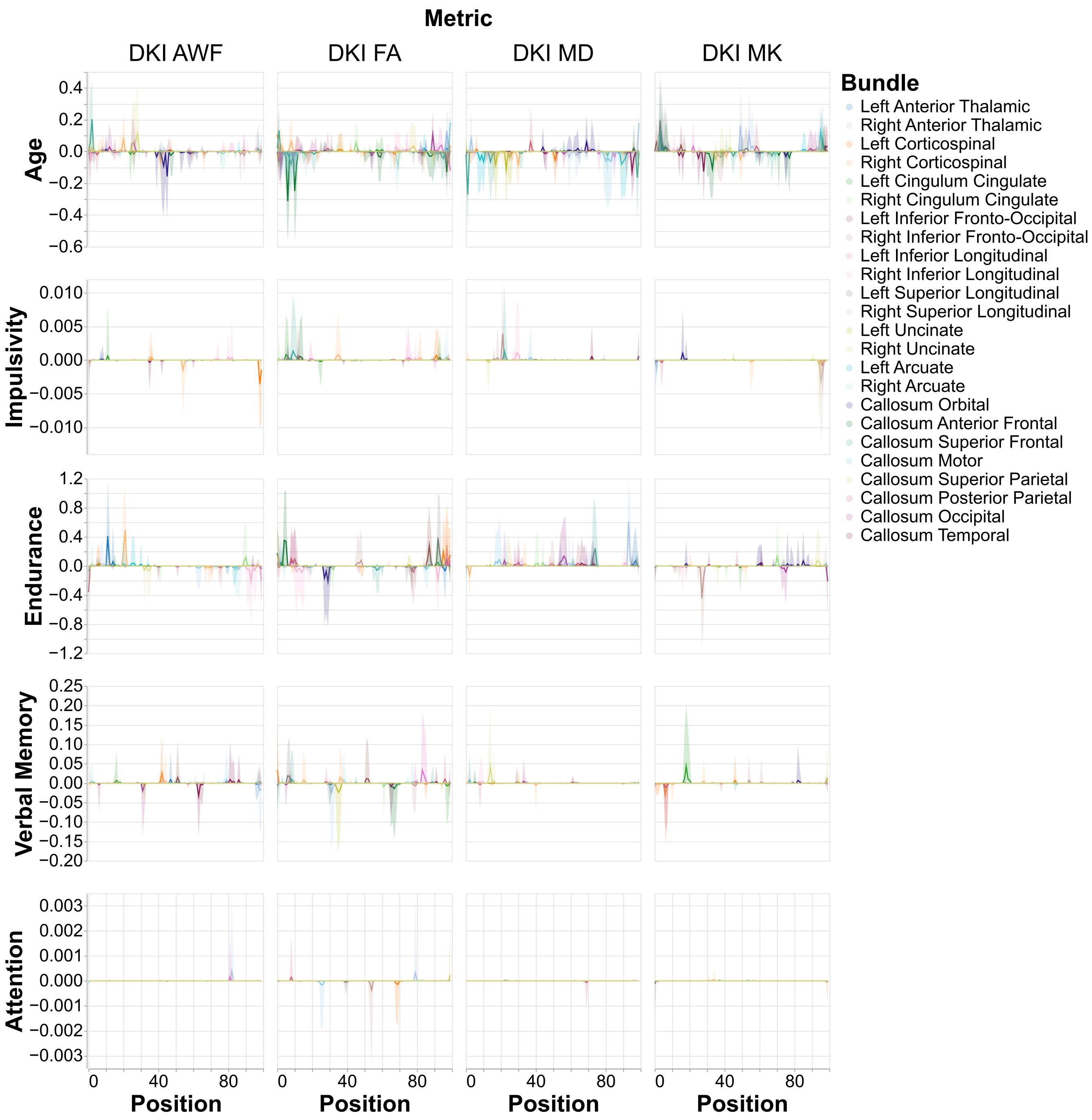

Figure S2. LASSO weights for prediction of the last five phenotypes, trained using tract profiles. Solid lines show the mean model weight across bootstraps for every tract, across every node, and the shaded area show the 95% confidence intervals of the model weights. Different columns show different white matter tissue properties, while the columns show five different phenotypes.

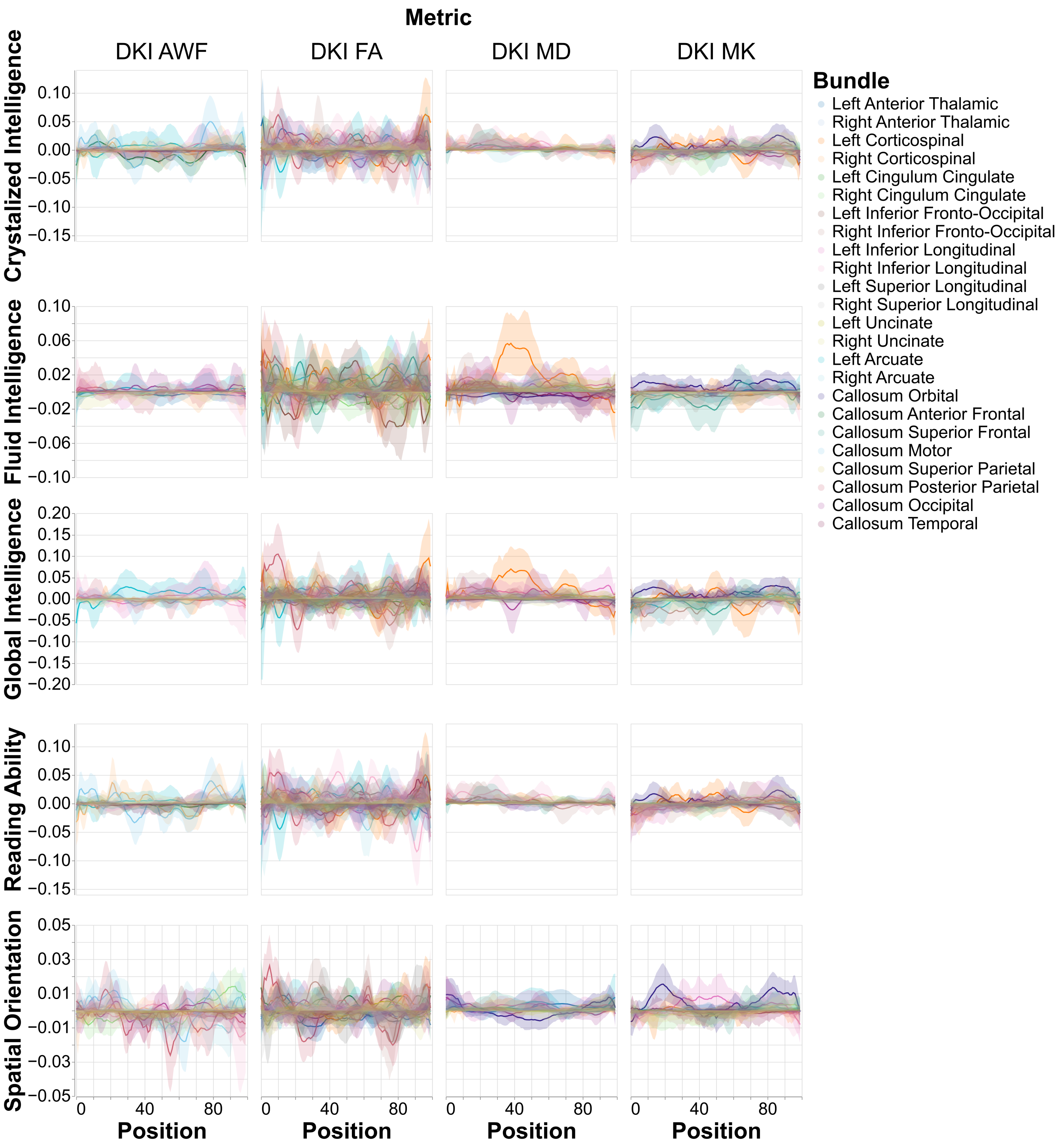

Figure S3. SGL weights for prediction of the first five phenotypes, trained using tract profiles. Solid lines show the mean model weight across bootstraps for every tract, across every node, and the shaded area show the 95% confidence intervals of the model weights. Different columns show different white matter tissue properties, while the columns show five different phenotypes.

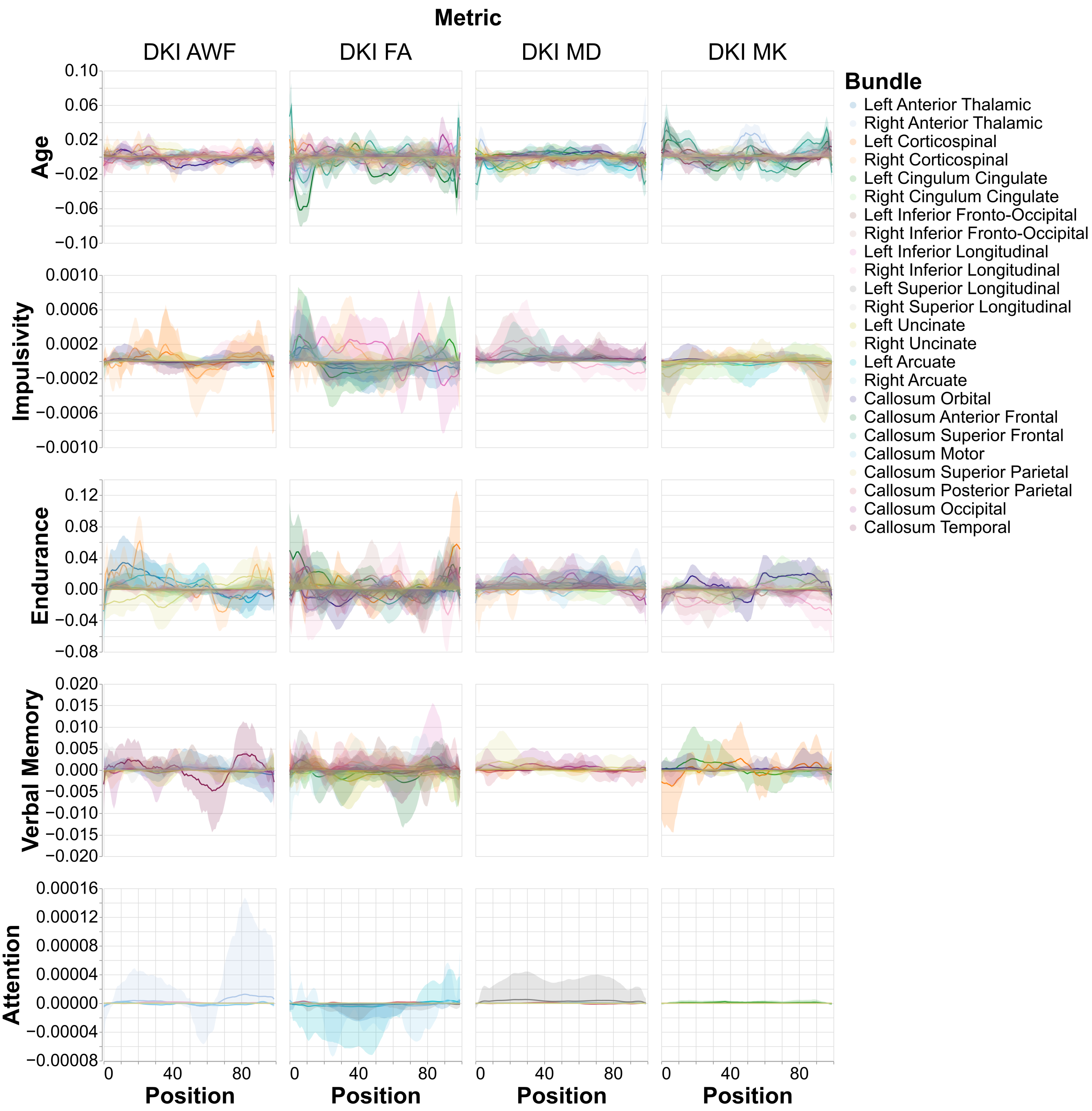

Figure S4. SGL weights for prediction of the last five phenotypes, trained using tract profiles. Solid lines show the mean model weight across bootstraps for every tract, across every node, and the shaded area show the 95% confidence intervals of the model weights. Different columns show different white matter tissue properties, while the columns show five different phenotypes.
